# Vasopressin for the treatment of vasodilatory shock: an ESICM systematic review and a meta-analysis

**DOI:** 10.1186/cc9512

**Published:** 2011-03-11

**Authors:** A Polito, E Parisini, Z Ricci, S Picardo, D Annne

**Affiliations:** 1 Ospedale Pediatrico Bambino Gesu, Roma, Italy; 2 Italian Institute of Technology, Milan, Italy; 3 Hôpital Raymond Poincaré (Assistance Publique-Hôpitaux de Paris), Garches, France

## Introduction

We examine benefits and risks of vasopressin/terlipressin use in patients with vasodilatory shock on mortality and morbidity.

## Methods

We searched the CENTRAL, MEDLINE, Embase, and LILACS (through to August 2010) databases. Randomized and quasi-randomized trials of vasopressin/terlipressin versus placebo or supportive treatment in adult and pediatric patients with vasodilatory shock were included. The primary outcome for this review was short-term all-cause mortality.

## Results

**Figure 1:**
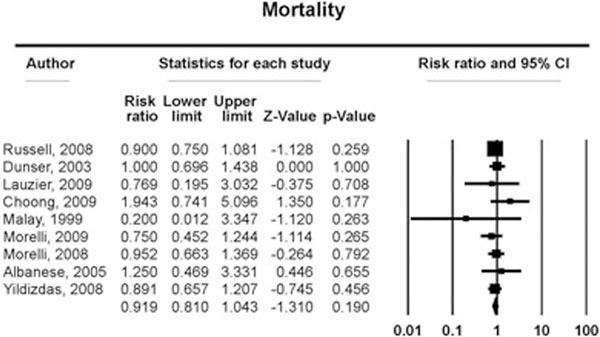
**Overall mortality**.

**Figure 2:**
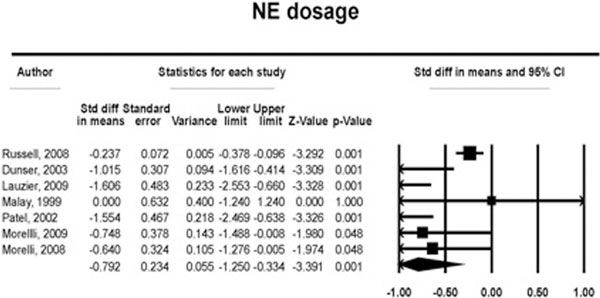
**Norepinephrine dosage**.

We computed data from 10 randomized trials (*n *= 1,111). The overall (28-day, 30-day, ICU, hospital and 24-hour) mortality for those treated with vasopressin and terlipressin versus control patients was 237 of 582 (40.7%) versus 226 of 528 (42.8%) (RR, 0.92; 95% CI, 0.81 to 1.04; *P *= 0.19; *I*^2 ^= 0%) without increasing the risk of AEs (nine trials 59/585, 10.0% vs. 55/529, 10.3%) (RR, 1.81; 95% CI, 0.62 to 1.86; *P *= 0.78; *I*^2 ^= 0%). See Figure 1. Patients receiving vasopressin/terlipressin are associated with a lower dosage of norepinephrine (seven trials, -0.79 μg/kg/minute (95% CI, -1.25 to -0.33; *P *< 0.001; *I*^2 ^= 73.6%) and a trend towards a higher urine output within 24 hours of treatment (six trials, 0.40 ml/kg/hour (95% CI, -0.11 to -0.92; *P *= 0.12; *I*^2 ^= 67.7%). See Figure 2.

## Conclusions

No significant effect of vasopressin/terlipressin therapy on all-cause mortality was demonstrated. Overall, there is no evidence to support the routine use of vasopressin or terlipressin in the management of patients with vasodilatory shock. There was, however, a reduction in the dose of norepinephrine used for those patients receiving vasopressin/terlipressin.

